# Poisoning by Urine

**Published:** 1846-02

**Authors:** G. F. Collier


*Poisoning by Urine.* By G. F. Collier, Esq., M. D.—I present to the public through the medium of "The Lancet," the following curious and instructive case. It abounds with matter for reflection, and it is especially worthy the consideration of those chemical physicians of the present time who are enthusiastically sanguine in their expectations of discovering the "fons et origo mali," and, by consequence, the curative or palliative indications, by reference to a patients secretions; and who confidently pronounce on the abnormal wear and tear of a man's cerebrum, by noting the morbid excess of phosphorus (a cerebral element) in his urine.

The case becomes additionally interesting, and, as I think, valuable, by the circumstance of its having been tested by the lapse of eight years. I shall offer no theories, no conjectures, I think it speaks for itself.

December 13th, 1838.—Thomas P——, of Turnham green, aged thirty-four, a day-laborer working on the roads, presented himself for advice, having for some days been afflicted with a dropsy. His face is very much swollen; and the anasarca generally more prominent in the upper, than in the lower, parts of the body. He says, that until now he scarcely knows what it is to be ill; that at, and since, the fall of the leaf he has had a troublesome eruption to which he had been many years occasionally subject; as it lasted longer than usual, he was advised by an old woman to drink his own urine for nine days, taking early in the morning, on a fasting stomach, precisely the whole quantity voided on going to bed; says he got through the nine doses, but thinks he began to swell before he had finished the course; he has fluid in the abdomen, and the parietes of chest and belly, swelling, however, greatest in the upper parts; the urine is scanty, thick, deep brown, and very offensive; he has a heaviness of the head, increased on stooping, and his own words are, that he feels too heavy for work, having neither his usual warmth nor life in him; he has little thirst; his pulse is under 90; his countenance pale, and its expression heavy and vague; he walks with a stick because, he says, he feels the want of support; he has not been unusually exposed to cold or to wet, and is of temperate habits; the urine is not albuminous; he loses his breath if he walks quick, and is obliged to stop. Deeming this case to be due to the poisonous impression, or to the ingestion of the urine by endosmosis into the circulation, I ordered a smart purgative of calomel and colocynth, and ofterwards six pills of calomel, squill, and digitalis, with heath broom tea. The water was all dispersed, and the man back to his work within ten days; he required no repetition of the medicine; the urine did not pass as it was used to do before this illness, till the ninth day.—*Ibid.*

